# Pheochromocytoma in a Twelve-Year-Old Girl with SDHB-Related Hereditary Paraganglioma-Pheochromocytoma Syndrome

**DOI:** 10.1155/2014/273423

**Published:** 2014-08-19

**Authors:** Daryl Graham, Megan Gooch, Zhan Ye, Edward Richer, Aftab Chishti, Elizabeth Reilly, John D'Orazio

**Affiliations:** ^1^Department of Pediatrics, University of Kentucky College of Medicine, Lexington, KY 40536, USA; ^2^University of Kentucky College of Medicine, Lexington, KY 40536, USA; ^3^Department of Pathology, University of Kentucky College of Medicine, Lexington, KY 40536, USA; ^4^Department of Radiology, University of Kentucky College of Medicine, Lexington, KY 40536, USA; ^5^Markey Cancer Center, University of Kentucky College of Medicine, Combs Research Building, 800 Rose Street, Lexington, KY 40536-0096, USA

## Abstract

A twelve-year-old girl presented with a history of several weeks of worsening headaches accompanied by flushing and diaphoresis. The discovery of markedly elevated blood pressure and tachycardia led the child's pediatrician to consider the diagnosis of a catecholamine-secreting tumor, and an abdominal CT scan confirmed the presence of a pheochromocytoma. The patient was found to have a mutation in the succinyl dehydrogenase B (SDHB) gene, which is causative for SDHB-related hereditary paraganglioma-pheochromocytoma syndrome. Herein, we describe her presentation and medical management and discuss the clinical implications of SDHB deficiency.

## 1. Case Presentation

A previously healthy 12-year-old girl presented with worsening headaches of six-month duration, described as diffuse, throbbing, and intense, occurring daily and lasting up to eight hours or more. They were not associated with time of day, posture, activity, and prodromal visual, auditory, or gustatory symptoms, and there were no associated neurologic abnormalities. Headaches were occasionally accompanied by lightheadedness, nausea, palpitations, and flushing. She had lost roughly 25% of her preillness body weight, had been fatigued and “warm,” and noted polydipsia and polyuria for several days prior to admission. The patient's mother, an experienced cardiology nurse, reported that the patient's blood pressure had been as high as 200/160 mmHg. The patient had a history of prematurity, being born at 27-week gestation and requiring intubation and mechanical ventilation for prematurity-induced respiratory distress. However, aside from exercise-induced reactive airways disease, she had no other ongoing medical problems. The patient had normal growth and development and was doing well in school. Family history was noncontributory for oncologic or endocrine diseases.

At her primary care physician's office, the patient's blood pressure was 205/160 mmHg, and the heart rate was 120 beats per minute. An abdominal CT scan, ordered to investigate the possibility of a neuroendocrine tumor, revealed a large right-sided suprarenal mass, which prompted a referral to our institution for further workup and management. The patient was referred to our service with the presumptive diagnosis of malignant hypertension secondary to a catecholamine-secreting pheochromocytoma (PCC) of the right adrenal gland. On arrival, the patient was alert, well-appearing, and in no distress. She was well-developed and nondysmorphic. The height was 152 cm (25th percentile for age) and weight was 54 kg (75th percentile for age). She was afebrile, the respiratory rate was 20 breaths per minute, the heart rate was 124 beats per minute and regular, and the blood pressure was 157/107 mmHg (the 95th percentile for a girl her age and height is 123/80 mmHg). Oxygen saturation was >95% on room air. She was mildly diaphoretic but had normal skin turgor and perfusion. Capillary refill was brisk and she was slightly flushed in her cheeks. Skin examination revealed no rashes, abnormal lesions, growths, café-au-lait spots, or axillary freckling. Cardiac examination revealed a hyperdynamic precordium and a grade II/VI systolic murmur best heard at the upper left sternal border. Peripheral pulses were 2+ throughout and all extremities were warm and well-perfused. The abdomen was soft, nontender, and nondistended without palpable masses or organomegaly. The patient was alert and oriented to person, place, and time. The neurologic and musculoskeletal exams were nonfocal.

**Figure 1:**
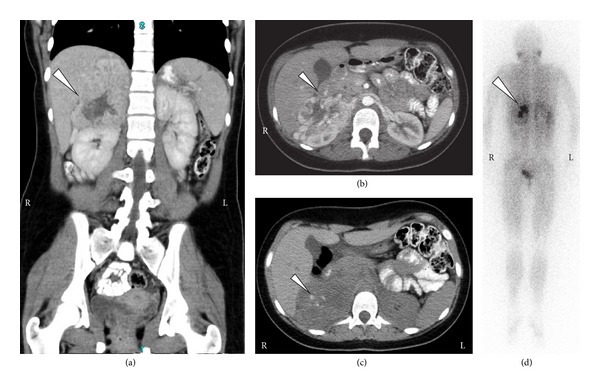
Radiologic imaging of the patient at presentation. (a, b, c) Computed tomography images showing a right-sided contrast-enhancing suprarenal mass (white arrows) displacing both the kidney and the liver. Note calcifications in the mass (arrow; image (c)), which is typical for pheochromocytoma. (d) MIBG nuclear scan showing uptake by the adrenal mass (arrow) and no evidence of other sites of disease.

**Figure 2:**
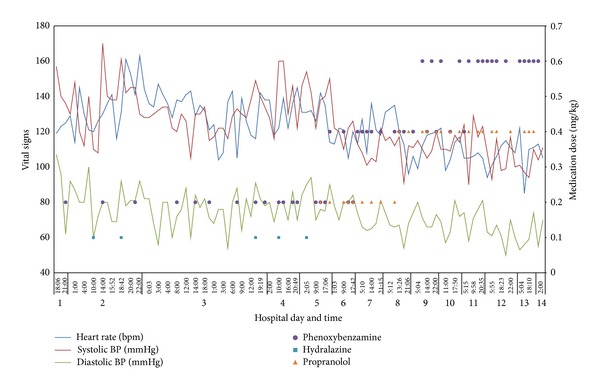
Sympathetic blockade and vital signs. Clinical data showing heart rate, systolic blood pressure, and diastolic blood pressure from admission until the time of tumor resection. Also shown are anticatecholamine medications and their doses over time. Note the progressive decreases in hypertension and tachycardia over the patient's hospital course.

**Figure 3:**
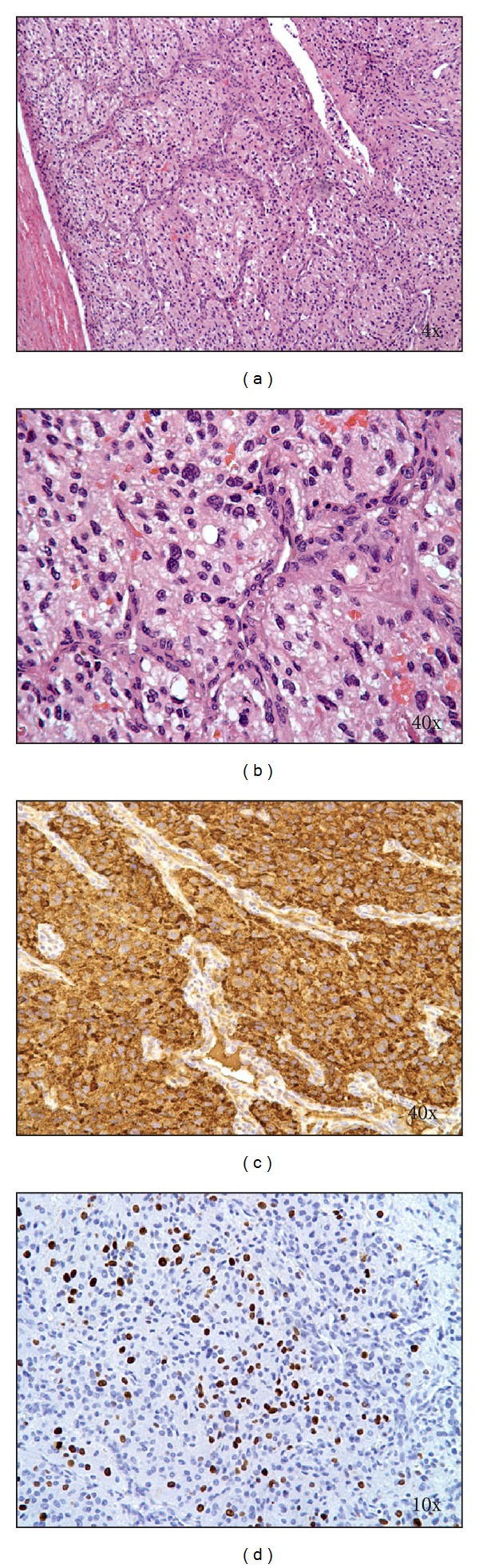
Pathologic images of resected tumor. (a, b) H & E-stained sections of the tumor showing tumor cells with abundant eosinophilic cytoplasm arranged in a nested and trabecular pattern and surrounded by fibrovascular stroma. (c) Chromogranin A staining demonstrating strong positivity of tumor cells. (d) Ki67 (MIB-1) immunostaining confirmed a high proliferation index (>10%) of the tumor cell population.

The patient's CBC, differential, peripheral blood smear, electrolytes, and liver function tests were all normal except for serum glucose of 125 mg/dL (normal 60–99 mg/dL). Serum uric acid was mildly elevated at 7.0 mg/dL (2.3–5.9 mg/dL), and serum lactate dehydrogenase (LDH) fell within the normal range (201 U/L; normal 110–293 U/L). Ionized calcium, serum cortisol, and thyroid stimulating hormone (TSH) were normal. Serum and urine catecholamines were markedly abnormal, especially plasma norepinephrine and urine normetanephrine (Table 1). Radiographic imaging confirmed a large right-sided suprarenal mass (Figure 1). The patient was diagnosed with malignant hypertension secondary to PCC. She was placed on telemetry and was started on intravenous fluids and *α*-blockade with phenoxybenzamine; headaches, flushing, diaphoresis, and nausea resolved within 24 hours. The patient was treated with progressive *α* and *β* catecholamine blockade (Table 2); the patient's medication history and vital findings are summarized (Figure 2). The tumor was completely resected on the fifteenth day of catecholamine blockade. The patient tolerated anesthesia well, and the blood pressure and heart rate remained stable. The patient's postoperative course was complicated by rebound hypotension requiring robust fluid support which led to pulmonary edema and respiratory failure requiring mechanical ventilation and pressor support. The patient recovered and was discharged 10 days after tumor resection. Histologic examination of the resected tumor confirmed that it was a PCC (Figure 3).

Currently, the patient is well now nine months after diagnosis. She is symptom-free and has normal blood pressure and plasma catecholamines. Because of the strong association between PCC and inherited cancer syndromes, the patient was evaluated for known genetic mutations associated with PCC and PGL (paraganglioma). The NF1, RET, TMEM127, VHL, MAX, and succinate dehydrogenase (SDHx) genes were sequenced, revealing a p.R27∗ point mutation in SDHB (c.79C>T) resulting in the change of a cytosine to a thymine and creating a premature stop codon in exon 2. This mutation, known to predispose to PGLs and PCCs, also increases risk for renal cell carcinoma [1–8].

Because of the risk of relapse and/or development of a secondary malignancy, we have educated the patient and her family about signs/symptoms that might indicate disease recurrence (e.g., headache, hypertension, flushing, diaphoresis, etc.). Following National Comprehensive Cancer Network (NCCN) guidelines, our surveillance plan includes a careful history and physical, blood pressure measurement, and urine and plasma catecholamine assessment every three months through the first year and every 6–12 months thereafter through 10 years. We will incorporate abdominal ultrasonography and/or MRI at her clinic visits as well as yearly FDG-PET scans. Critically, we have referred her immediate family for formal genetic evaluation to screen for SDHB mutations and to provide genetic counseling regarding the implications of the patient's identified cancer syndrome.

## 2. Discussion

PCCs and PGLs are neuroendocrine tumors that arise from chromaffin cells in the adrenal gland (PCC) or in extra-adrenal sympathetic nerve ganglia (PGL). Because of their chromaffin cell origin, PCCs secrete catecholamines, which explains the underlying sympathetic paraneoplastic symptoms many patients exhibit. However, unlike normal chromaffin cells which are innervated by sympathetic neurons and release catecholamines only when initiated by an appropriate neural impulses, PCCs produce and release catecholamines in an unregulated and markedly exaggerated way. As with our patient, PCCs frequently come to medical attention because of symptoms of catecholamine excess rather than because of problems caused by the tumor itself. Most PCCs, including those presenting in childhood, preferentially secrete norepinephrine but they can also secrete epinephrine and dopamine. Catecholamines mediate their effects through *α*-adrenergic and *β*-adrenergic receptors. Alpha effects include arteriolar constriction causing hypertension and diminished insulin secretion and enhanced gluconeogenesis and glycogenolysis all of which promote hyperglycemia. Beta stimulation, on the other hand, enhances cardiac contractility and rate. The most common presenting signs and symptoms, therefore, include tachycardia, hypertension, palpitations, diaphoresis, flushing, and headache. Weight loss is common because of a chronic hypermetabolic state. Early identification is critical to prevent secondary complications including myocardial infarction, cerebrovascular accident, renal injury, and arterial aneurysms. Other conditions to consider in the differential diagnosis include neuroblastoma, coarctation of the aorta, and other causes of hypertension. Diagnosis of PCCs relies on detection of elevations of catecholamines and metanephrines in plasma and/or the urine. Plasma metanephrine testing is preferred to urine testing. Radiologic imaging localizes and confirms tumor presence. Beside conventional imaging modalities such as computed tomography (CT) or magnetic resonance imaging (MRI), nuclear medicine approaches such as FDG-PET scanning can be helpful in tumor localization and staging. DOPA-PET scanning is highly specific to catecholamine-producing tumors and therefore can be useful in differentiating PCC/PGL from other metabolically active lesions such as inflammatory or infectious lymph nodes [9, 10]. Alternatively, because cells in sympathetic neurons preferentially take up metaiodobenzylguanidine, MIBG scanning is used in certain centers to determine anatomic sites of PCC involvement.

The annual incidence rate for PCCs and PGLs is roughly 1 in 100,000 persons, with up to 20% occurring in the pediatric age range. Among affected children, 70% of cases are unilateral, are localized to the adrenal gland, and are presenting between the ages of 6 and 14 years with a mean of 11 years. The majority of PCCs diagnosed in children are benign, with tumors remaining localized to their site of origin and cured by surgical resection alone [11]. Because of unregulated sympathetic release, PCCs can precipitate serious comorbid conditions; therefore, timely diagnosis and management are important [12]. Caution must be taken in preparing patients with PCCs for tumor resection because of the risk of dramatic blood pressure swings. Catecholamine blockade before surgery is critical to avoid unpredictable and exaggerated catecholamine release at the time of tumor resection [13]. Perioperative mortality has dropped from as high as 40% to under 3% over the last several decades because of more effective preoperative catecholamine blockade, better diagnostic accuracy in localization, improved surgical skills, and better perioperative medical management [14, 15]. In general, preoperative medical management is accomplished by sequential *α*-blockade followed by *β*-blockade along with restoration of adequate circulating volume to replenish intravascular volume reduced from catecholamine-induced vasoconstriction. The most widely used alpha antagonist for PCCs is phenoxybenzamine, a nonselective alpha adrenergic blocker. In general dose is titrated to effect, up to 1 mg/kg/day [16]. Major side effects include reflex tachycardia and sedation, and the long half-life of the drug can lead to sustained hypotension after tumor resection. Selective alpha-1 antagonists such as prazosin and doxazosin may be associated with less reflex tachycardia; however, clinical studies comparing either to phenoxybenzamine have not been conclusive. Beta blockers, typically metoprolol or propranolol, are effective at preventing or treating catecholamine-induced tachyarrhythmias; however, their use as front-line agents is contraindicated until effective *α*-blockade has been established due to unopposed *α*-adrenergic stimulation by catecholamine excess [17].

PCCs and PGLs can occur sporadically or as part of an inherited predisposition syndrome, most notably multiple endocrine neoplasia (MEN) syndromes 2A and 2B, neurofibromatosis (NF) type 1, and von Hippel-Lindau (VHL) disease [18–22]. More recently, genes that encode proteins in the mitochondrial complex II, particularly the succinyl dehydrogenase components SDHD, SDHB, and SDHC, have been identified as causative for PCC-PGL syndromes [1, 23–27]. Roughly a third of PCCs occur in the setting of an inherited cancer syndrome and the likelihood of a global predisposition syndrome underlying a PCC or PGL is higher for children than adults [28, 29]. Our patient was found to harbor a p.R27∗ point mutation in SDHB (c.79C>T) creating a premature stop codon in exon 2 and establishing the diagnosis of SDHB-related hereditary paraganglioma-pheochromocytoma syndrome [30]. SDHB mutations, known to predispose to PGLs and PCCs, also increase risk of renal cell carcinoma [31]. Letouzé and coworkers recently reported that SDHx mutations lead to excess methylation and epigenetic silencing of key genes involved in neuroendocrine differentiation, thereby contributing to malignant degeneration of cells [32]. In one series studying patients with SDHB mutations, the mean age at diagnosis was 33.7 years with most patients presenting with extra-adrenal tumors [30]. On reviewing the literature, we found that PCC/PGLs associated with SDHB mutations have been described in children as young as eight years old; however, most pediatric SDHB-associated PCC/PGLs present in adolescence [33–35]. SDHB mutations are known to be highly penetrant, with lifetime risk of PGL/PCC as high as 77–100% and lifetime risk of renal cell carcinoma estimated to be 10–20% [35].

Overall, children with PCC have an excellent long-term prognosis. In one large-scale study, mean life expectancy was 62 years among patients with hereditary PCC/PGL; however, SDHB mutations are associated with more aggressive disease in terms of early age at diagnosis, higher metastatic potential, recurrence, and the development of other primary malignancies (PCC, PGL, and renal cell carcinomas) [36, 37]. As a result, our patient will be monitored closely including every three-month history and physical clinic visits accompanied by plasma chromogranin A and metanephrine testing along with abdominal ultrasonography. We plan yearly FDG-PET or DOPA-PET scans [38]. Perhaps most importantly, we have instructed the patient and her family to report signs of catecholamine excess (diaphoresis, tachycardia, flushing, headache, and hypertension) to us as rapidly as possible should they develop between clinic visits. Thus far, the patient is well, now almost nine months since her initial presentation, without evidence of disease. Genetic evaluation of other family members for SDHB mutations is in progress, along with genetic counseling regarding the implications of the patient's identified cancer syndrome. It is critical to evaluate patients for inherited cancer predisposition syndromes in order to understand what tumor(s) and other conditions the patient may be at risk for, to develop an appropriate surveillance plan to monitor the patient for sequelae specific to the particular genotype, to provide genetic counseling to the patient, and to offer genetic testing and counseling to the patient's family [39, 40].

## Figures and Tables

**Table 1:** Presenting serum and urine catecholamine levels.

Test	Patient result	Normal range
Plasma		
(i) Total catecholamines	>8,705 pg/mL	123–1,125 pg/mL
(ii) Norepinephrine	>8,000 pg/mL	112–1,109 pg/mL
(iii) Dopamine	653 pg/mL	10–20 pg/mL
(iv) Epinephrine	52 pg/mL	50–95 pg/mL

Urine		
(i) Total metanephrines	9,907 mcg	110–714 mcg
(ii) Normetanephrine	9,828 mcg	67–503 mcg
(iii) Homovanillic acid	66.1 mg	<6.8 mg
(iv) Metanephrine	79 mcg	<275 mcg

**Table 2:** Suggested algorithm for preoperative catecholamine blockade for PCC/PGL. Patients are best managed first with a pure alpha adrenergic blockade to relax arteriolar smooth muscles and reduce catecholamine-induced blood vessel constriction. Once the blood pressure is well-controlled, beta blockers can be added to treat tachycardia.

Medication	Recommended dose	Notes
Phenoxybenzamine	(i) Days 1-2: 0.2 mg/kg (max 10 mg) q12h (ii) Days 3-4: 0.2 mg/kg q8h (iii) Days 5-6: 0.2 mg/kg q6h (iv) Days 7-8: 0.4 mg/kg (max 20 mg) q6h (v) Days 9-10: 0.4 mg/kg qid (vi) Days 11-12: 0.6 mg/kg (max 30 mg/dose) q6h (vii) Days 13-14: 0.6–0.8 mg/kg q6h	(i) Goal is low normal blood pressure, at least below 50th percentile but preferably below 25th percentile (ii) Side effects: congestion, tachycardia, hypotension, dizziness, especially when standing up (and true orthostatic hypotension), malaise, anorexia, and very rare allergic reactions (iii) Simultaneously load the patient with salt and water to allow maximum blockade

Beta blockade	Propranolol 0.5 mg/kg/dose tid and advanced as necessary to keep HR <110	(i) Should never be used as a first agent (ii) Helpful for symptomatic tachycardia (iii) Begin 5–7 days after phenoxybenzamine

Notes that if the timetable for surgery must be compressed (so that surgical resection is planned 7-8 days after diagnosis), we consider advancing catecholamine blockade daily and using metyrosine to reduce amount of catecholamines produced by the tumor.
